# Evaluation of the Hepa Wash^®^ treatment in pigs with acute liver failure

**DOI:** 10.1186/1471-230X-13-83

**Published:** 2013-05-13

**Authors:** Ahmed Al-Chalabi, Edouard Matevossian, Anne-K v Thaden, Peter Luppa, Albrecht Neiss, Tibor Schuster, Zejian Yang, Catherine Schreiber, Patrick Schimmel, Ewald Nairz, Aurel Perren, Peter Radermacher, Wolfgang Huber, Roland M Schmid, Bernhard Kreymann

**Affiliations:** 1 II Medizinische Klinik, Klinikum rechts der Isar, Technische Universität München, München 81675, Gremany; 2 Chirurgische Klinik und Poliklinik, Klinikum rechts der Isar, Technische Universität München, München, Germany; 3 Zentrum für Präklinische Forschung, Klinikum rechts der Isar, Technische Universität München, München, Germany; 4 Institut für Klinische Chemie und Pathobiochemie, Klinikum rechts der Isar, Technische Universität München, München, Germany; 5 Clinrex GmbH, Munich, Muchen, Germany; 6 Hepa Wash GmbH, Munich, Munchen, Germany; 7 Institute of Medical Statistics and Epidemiology, Technische Universität München, Munchen, Germany; 8 Institute of Pathology, Universtiy of Bern, Bern, Switzerland; 9 Institute of Pathology, Technical University Munich, Munchen, Germany; 10 Sektion Anästhesiologische Pathophysiologie und Verfahrensentwicklung, Universitatsklinikum, Ulm, Germany

**Keywords:** Artificial liver, Acute liver failure, Albumin dialysis, Animal model, Swine, Renal dialysis, Multiple organ failure, Capillary leak syndrome, Cardiovascular failure, Renal failure

## Abstract

**Background:**

Mortality of patients with acute liver failure (ALF) is still unacceptably high. Available liver support systems are still of limited success at improving survival. A new type of albumin dialysis, the Hepa Wash^®^ system, was newly introduced. We evaluated the new liver support system as well as the Molecular Adsorbent Recycling System (MARS) in an ischemic porcine model of ALF.

**Methods:**

In the first study animals were randomly allocated to control (n=5) and Hepa Wash (n=6) groups. In a further pilot study, two animals were treated with the MARS-system. All animals received the same medical and surgical procedures. An intraparenchymal intracranial pressure was inserted. Hemodynamic monitoring and goal-directed fluid therapy using the PiCCO system was done. Animals underwent functional end-to-side portacaval shunt and ligation of hepatic arteries. Treatment with albumin dialysis was started after fall of cerebral perfusion pressure to 45 mmHg and continued for 8 h.

**Results:**

All animals in the Hepa Wash group survived the 13-hour observation period, except for one that died after stopping treatment. Four of the control animals died within this period (p=0.03). Hepa Wash significantly reduced impairment of cerebral perfusion pressure (23±2 vs. 10±3 mmHg, p=0.006) and mean arterial pressure (37±1 vs. 24±2 mmHg, p=0.006) but had no effect on intracranial pressure (14±1 vs. 15±1 mmHg, p=0.72). Hepa Wash also enhanced cardiac index (4.94±0.32 vs. 3.36±0.25 l/min/m2, p=0.006) and renal function (urine production, 1850 ± 570 vs. 420 ± 180 ml, p=0.045) and eliminated water soluble (creatinine, 1.3±0.2 vs. 3.2±0.3 mg/dl, p=0.01; ammonia 562±124 vs. 1382±92 μg/dl, p=0.006) and protein-bound toxins (nitrate/nitrite 5.54±1.57 vs. 49.82±13.27 μmol/l, p=0.01). No adverse events that could be attributed to the Hepa Wash treatment were observed.

**Conclusions:**

Hepa Wash was a safe procedure and improved multiorgan system failure in pigs with ALF. The survival benefit could be the result of ameliorating different organ functions in association with the detoxification capacity of water soluble and protein-bound toxins.

## Background

Since the introduction of renal replacement therapy, different methods of extracorporeal liver support therapies were tested. They appear to improve the survival of patients with acute liver failure but didn’t have a major impact on survival of patients acute-on-chronic liver failure [1]. The mortality of liver failure remains unacceptably high and liver transplantation is still the only effective treatment. Albumin dialysis is one of the latest therapeutic approaches introduced. To date there are two types of albumin dialysis: Single Pass Albumin Dialysis (SPAD) and Molecular Adsorbent Recycling System (MARS^®^). The former has been described mainly in sporadic cases, where its use is limited by the costs of albumin and efficacy [2-6]. MARS has been used more widely and it has been shown to be effective in improving biochemical profile and hepatic encephalopathy as well as survival in specific patient groups [7-9]. A hard evidence of survival benefit of the MARS procedure is however still lacking, as the results of a large multicenter study have shown no improvement of mortality in patients with fulminant and subfulminant liver failure [10]. Different bioartificial liver support systems were used enthusiastically in small clinical trials [11-13] but in a multicenter study conducted by Demetrieu et al., they were found to improve the survival of patients with fulminant/subfulminant liver failure only after excluding primary nonfunction following liver transplantation which was included in the study group [14]. Finally, extracorporeal therapy with fractionated plasma Separation and Adsorption (Prometheus^®^) was associated with an improved survival in patients with acute liver failure, but mainly in retrospective and case report studies [15-17].

Animal models simulating acute liver failure (ALF) are not only needed to study the underlying poorly understood pathophysiological mechanisms but are also important for the evaluation of new liver support systems prior to introduction in clinical studies. Devascularizing ALF is one of the most common animal models used for evaluating liver support systems. Recently, our group developed such a model and tested its reproducibility [18].

**Figure 1:**
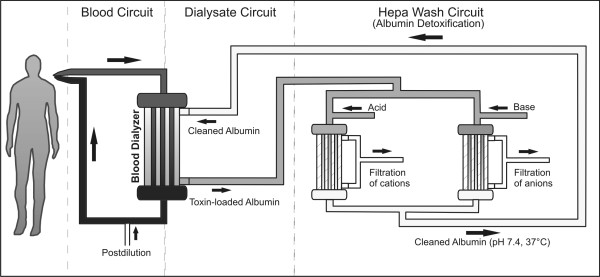
**A schematic representation of Hepa Wash. **The new liver support system is composed of three circuits: the blood, dialysate and Hepa Wash circuits.

In this work we present a preclinical study that evaluates the safety and efficacy of a new liver support system (Hepa Wash^®^, Figure 1), a type of albumin dialysis, in a large animal model of ischemic ALF. We also present the results of the MARS treatment in two pilot animals of the same model.

## Methods

### Animals and housing

**Figure 2:**
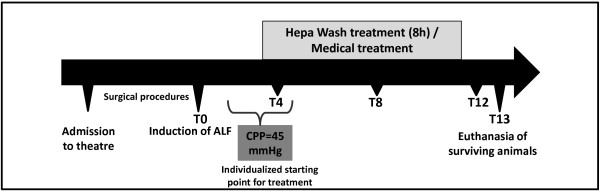
A timeline of the experiments.

The preclinical study was approved by the ethical committee for animal studies in Bavaria, Germany. German landrace female pigs (~60 kg) were procured from the animal farm and were kept in the animal housing in the center for preclinical research in the hospital rechts der Isar for about 4–7 days to allow for accommodation. Animals were fasted approximately twelve hours before the operation but had free access to water. Housing and all medical and surgical procedures (Figure 2) were in accordance with the national animal protection act (Tierschutzgesetz) and the institution guidelines (registration number 55.2-1-54-2531-60-07, approved on 20.6.2007). Experiments were performed between January and April 2008. The Animals were divided randomly into two groups: the control (n=6) and the Hepa Wash (n=6) groups. One animal in control group was excluded due to insufficient induction of liver failure (which was histopathologically confirmed).

In a small pilot study (n=2) that was conducted independently from the above randomized study, animals with acute liver failure were treated by the Molecular Adsorbent Recirculating System (MARS).

All animals in the Hepa Wash and the control groups and the two animals in the MARS pilot study were subjected to the same following surgical and medical procedures and all were treated under the same conditions e.g. noise.

### Anesthesia and ventilation

Premedication of the animals was performed with ketamine (15 mg/kg), azaperone (2 mg/kg) and atropine (0.5-1 mg/kg) intramuscularly, respectively. Induction of anesthesia with propofol (60–100 mg, i.v.) was followed by intubation. Anesthesia was maintained intravenously with propofol (up to 8.5 mg/kg/h) fentanyl (up to 0.015 mg/kg/h), and atracurium (up to 0.7 mg/kg/h) depending on the depth of anesthesia. Animals were ventilated (Cicero EM, Dräger Medical Deutschland GmbH, Lübeck, Germany) with intermittent positive pressure and a mixture of oxygen and air (FiO_2_ 30-60%). Mean airway pressure was aimed below 30 cmH2O and I:E ratio was adjusted to 1:2. The ventilation rate and tidal volume were set between 14–20 breaths per minute and 6–10 ml/kg, respectively, to ensure proper oxygenation.

Adequacy of anesthesia was assessed clinically by observing the animals ensuring that they have sufficient relaxation and analgesia. Spontaneous breathing must be absent.

### Cannulation

Prior to induction of ALF, two jugular veins were cannulated to facilitate fluid and drug infusions. Blood arterial pressure was measured invasively using a cannula inserted into the carotid artery. A dialysis catheter (13F high flow two-lumen 20 cm, Achim Schulz-Lauterbach VMP, Iserlohn, Germany) was inserted so that the tip is in the right atrium of the heart. The femoral artery was catheterized with a 5-French thermistor-tipped catheter (PV 2015L20, Pulsion Medical Systems AG, Munich, Germany). A surgically inserted suprapubic bladder catheter was used to collect urine. An intracranial pressure transducer, Neurovent-P Temp (Raumedic AG, Helmbrechts, Germany) was inserted and positioned in the cerebral parenchyma.

### Establishment of ALF

The surgical anatomy and procedures were described in more detail previously [18]. Laparotomy was performed and the structures in the hepatoduodenal ligament were exposed. The portal vein and inferior (caudal) vena cava were then partially clamped before a functional end-to-side portacaval shunt was established with polypropylene (prolene^®^, Ethicon Inc., Norderstedt, Germany). The development of splanchnic congestion was avoided by ensuring an adequate portal flow during partial clamping. All arteries supplying the liver and the hepatoduodenal ligament (except for bile duct which was left intact) were ligated (Vicryl^®^ 2/0, Ethicon Inc., Norderstedt, Germany). Cefuroxime 500 mg was administered as an infusion during surgery.

### Acid–base household and electrolytes

Sodium bicarbonate administration (8.4%) was administered to treat metabolic and respiratory acidosis if pH<7.3 or to increase bicarbonate levels (aim 28–30 mmol/l). Hyperkalemia was treated by insulin injections (5–25 IU) in boluses with simultaneous adjustment of glucose infusions. We used potassium-containing solutions or added KCl (20–80 ml, 1M) to the glucose infusions to correct hypokalemia. Hypocalcemia was treated by infusing calcium gluconate 10% 10-100ml/h.

### Fluid therapy

Infusion of crystalloids (saline, glucose 5% or 20%) was directed according to measurements of the PiCCO system (Pulsion Medical Systems AG, Munich, Germany). Aim was to keep extravascular lung water index (ELWI)<12 ml/kg and global end-diastolic volume index (GEDI) between 500 and 800 ml/m^2^ as possible. The choice of crystalloids was dependent on the electrolyte balance, glucose level and acid–base balance. We targeted a mean blood glucose level of around 120 mg/dl [19].

### Albumin dialysis

A laboratory prototype (Hepa Wash GmbH, München, Germany) was used to conduct the the Hepa Wash treatments. It is composed of three circuits: the blood, dialysate and Hepa Wash circuits (Figure 1). Approximately 40 grams of human serum albumin were used for each treatment. The composition of albumin dialysate in the hemodialyzers is similar to that of dialysate used in conventional hemodialysis (apart from containing 2% albumin). Postdilution 2l/h was performed with PrismaSol2^®^ (Gambro Hospal GmbH, Gröbenzell, Germany). In the Hepa Wash circuit, albumin dialysate is divided into two parts. Each part undergoes a change of pH value by adding acid or base before passing through the filters resulting in a release of albumin-bound toxins. The unbound portion of toxins is removed by a filtration process. The acidified and the alkalinized albumin dialysates join each other so that a physiological pH (range 6.9-7.6) is generated before passage in the dialysate compartment of the hemodialyzers.

The treatment sessions in the two animals treated with MARS were performed independently by the Hepanet GmbH, Hannover, an established provider of the MARS therapy. The system was filled with 100g albumin.

Heparin was given to all animals (including the control group) by continuous infusion. Anticoagulation was monitored through the activated clotting time (ACT), which was measured by the Hemochron^®^ (ITC, Edison, NJ). The ACT was kept between 150–250 seconds.

Both Hepa Wash and MARS were started as soon as the cerebral perfusion pressure dropped to 45 mmHg and were all continued for 8 hours.

### Sampling

Blood gas analysis was performed frequently to ensure optimal and quick adjustment of certain parameters e.g. glucose (Rapidpoint^®^ 405, Siemens Health Care Diagnostics Inc., Eschborn, Germany). Blood samples were collected immediately before induction of ALF (T0) and every two hours thereafter. Samples for the measurement of nitrate/nitrite blood levels were frozen at -80°C and analyzed using the chemiluminescence method.

### Euthanasia and autopsy

Surviving animals were sacrificed with a lethal dose of pentobarbitone and KCl injected intravenously 13 hours after induction of ALF. Animals were considered dead if cerebral perfusion pressure ≤ 5 mmHg for 5 minutes. All animals were examined for signs of bleeding during autopsy.

### Statistics

For comparing Hepa Wash with the control group, we used the cerebral perfusion pressure as the primary end point. The “ordered” hypotheses method was employed to avoid correction for the multiplicity of alpha error when multiple comparisons are performed [20]. If some of the data especially at the end of the experiments were missing due to death of the animal, then they were assumed to be equal to the latest measured value (the last value carried forward method). Data were expressed as mean ± SEM (standard error of the mean) - unless stated otherwise (as median and range) - after handling of the missing data. Comparisons were carried out as follows: The data were compared first from the latest time point i.e. eight hours after decrease of cerebral perfusion pressure to 45 mmHg. If the null hypothesis (no difference of cerebral perfusion pressure between the two groups) was rejected, an earlier adjacent time point was analyzed. Comparisons are repeated for the time points going backwards until the values are not significant anymore. Further statistical data, graphs and p-values for other parameters including the results of MARS treatments were displayed for explorative purposes only and not to be taken as confirmatory evidence.

Data were documented and analyzed using IBM SPSS 19.0 for Windows^®^. A non-parametric test (Mann–Whitney U) was used to compare the readings and biochemical values between groups, whereas the log-rank test was employed to statistically evaluate survival differences. A two-tailed p-value less than 0.05 was considered to indicate statistical significance.

## Results

### Cerebral parameters

**Figure 3:**
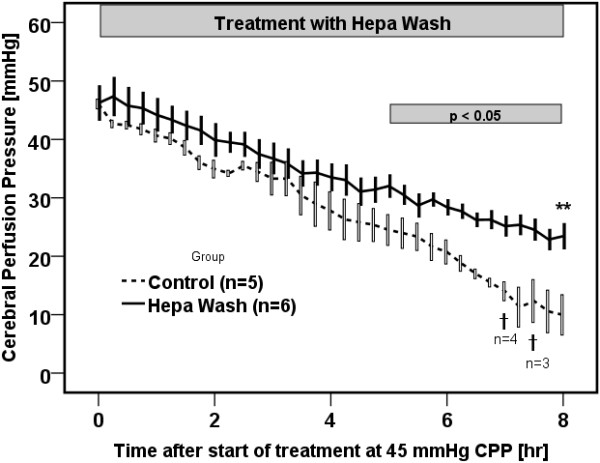
Hepa Wash significantly ameliorated cerebral perfusion pressure (CPP). * p<0.05 (difference between Hepa Wash and control); ** p=0.006 (difference between Hepa Wash and control); † Death of animals; SEM: standard error of mean.

The cerebral perfusion pressure decreased to 45 mmHg after a median of 3¾ h (2-5 h) in the Hepa Wash group, and after a median of 3¼ h (2-4½ h) in the control group from induction of liver failure (p=0.46). The cerebral perfusion pressure was significantly higher after 8 hours of treatment in the Hepa Wash group in comparison with the control group (23 ± 2 vs. 10 ± 3 mmHg, respectively, p=0.006) (Figure 3). A significant difference was found as early as five hours after reduction of cerebral perfusion pressure to 45 mmHg (p=0.045). In contrast, the intracranial pressure did not change significantly after 8 hours of treatment with Hepa Wash (14 ± 1 vs. 15 ± 1 mmHg, respectively, p=0.72) (Table 1).

**Table 1:** List of the main hemodynamic parameters of animals in the control and Hepa Wash groups

**Time [h]**	**T0**			**T4**			**T8**			**T12**		
**Group**	**Controls (n=5)**	**Hepa Wash (n=6)**	**MARS (n=2)**	**Control (n=5)**	**Hepa Wash (n=6)**	**MARS (n=2)**	**Control (n=5)**	**Hepa Wash (n=6)**	**MARS (n=2)**	**Control (n=2)**	**Hepa Wash (n=6)**	**MARS (n=1)**
Cerebral perfusion pressure [mmHg]	87 ± 10	90 ± 4	84, 70	40 ± 3	46 ± 3	35, 36	24 ± 1	34 ± 2*	21, 34	18, 6	22 ± 1*	10
Cardiac index [l/min/m2]	5.06 ± 0.30	5.13 ± 0.37	4.01, 4.5	5.15 ± 0.58	5.66 ± 0.50	5.65, 6.1	3.96 ± 0.17	5.36 ± 0.38*	5.37, 5.71	3.58, 2.51	4.94 ± 0.33*	-
ELWI [ml/kg]	8±1	7±1	6, 7	9±0	8±1	8, 7	9±1	7±1	7, 7	8, 13	8±1	-
GEDI [ml/m2]	598±28	620±31	578, 609	571±53	619±44	602, 579	524±58	626±28	653, 638	489, 476	622±41	-
SVRI [dyn.s.m2.cm-5]	1291±45	1361±200	1080, 1307	887±66	874±85	670, 660	815±69	741±71	539, 576	570, 762	607±49	-
Mean Arterial Pressure [mmHg]	96±9	99±4	93, 79	49±3	56±3	47, 46	38±2	48±1*	36, 48	29, 22	36±1*	34
Intracranial Pressure [mmHg]	9.3±0.8	9.1±0.9	8.2, 9.1	9.1±0.8	10.4±1.1	12.3, 10.4	13.6±1.9	13.9±2.3	14.7, 13.7	11.4, 16.4	13.9±0.8	24.4
Intracranial temperature [°C]	36.8±0.3	37.0±0.2	38, 37.9	36.9±0.2	37.4±0.3	37.6, 37.7	37.2±0.2	36.9±0.1	37.6, 37,3	36.5, 36.6	36.9±0.2	36.0

*ELWI*: extravascular lung water index. *GEDI*: global enddiastolic volume index. *SVRI*: systemic vascular resistance index.*p<0.05 (difference between Hepa Wash and control).

### Survival

**Figure 4:**
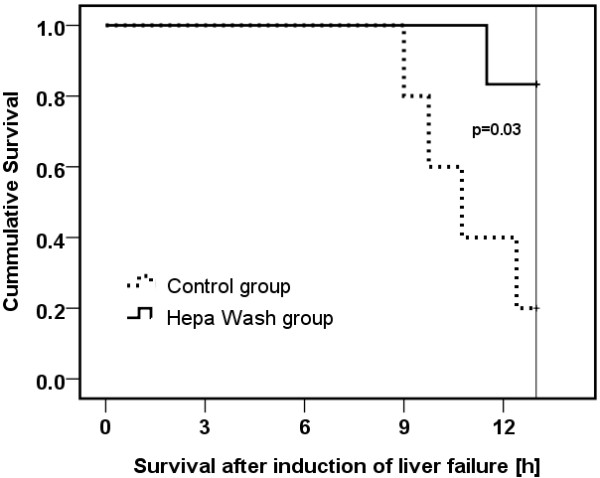
A cumulative survival analysis showing an improvement of survival with the Hepa Wash procedure.

Four animals in the control group died during the observation period with a median of 10¾ h (9-13 h) (Figure 4). Five animals in the Hepa Wash group survived the 13-hour observation period. Only one animal died in the Hepa Wash group, and this occurred shortly after the end of the 8-hour-treatment period with Hepa Wash. The corresponding log-rank test showed a significant survival improvement in the Hepa Wash group (p=0.03).

In contrast, the two animals in the MARS group survived for approximately 11½ and 12 h, respectively, after the induction of the liver failure. Deat h occurred after stopping treatment but also after a dramatic elevation of intracranial pressure shortly before end.

### Liver failure

Animals in the Hepa Wash and control groups showed signs of fulminant hepatic failure with a rapid worsening of Quick value (prothrombin time) (39 ± 4 vs. 33 ± 5% at T 12, respectively, p=0.71) and INR (2.0 ± 0.1 vs. 2.7 ± 0.8 at T12, respectively, p=0.57). Fibrinogen also decreased rapidly (218 ± 18 vs. 233 ± 54 mg/dl at T12, respectively, p=0.71).

### Hemodynamics

The mean arterial pressure in the Hepa Wash group was higher than in the control group (37 ± 1 vs. 24 ± 2 mmHg, respectively, p=0.006) after 8 hours of treatment (i.e. eight hours after cerebral perfusion pressure reached 45 mmHg) (Figure 3). The difference started to be significant after 4 hours of treatment (p=0.045) (Figure 3). The cardiac index was also higher in the Hepa Wash group at T12 (4.94 ± 0.33 vs. 3.36 ± 0.25, p=0.006). (Table 1). GEDI and ELWI were not significantly different between the two groups (Table 1). There was a positive fluid balance of around 230 ± 35 ml/h in the control group, whereas it was 320 ± 100 ml/h in the Hepa Wash group (p=0.46).

### Urine output

Animals in the Hepa Wash group produced more urine (1850 ± 570 ml) than in the control group (420 ± 180 ml), collected after cerebral perfusion pressure decreased to 45 mmHg (p = 0.045).

### Detoxification

**Figure 5:**
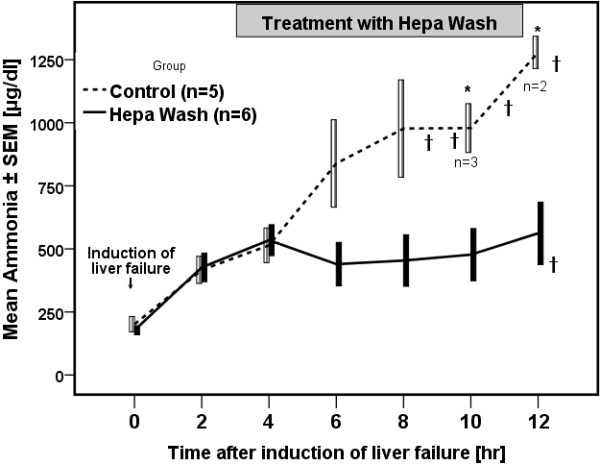
Elimination of ammonia by the Hepa Wash procedure. * p<0.05 (difference between Hepa Wash and control); † Death of animals; SEM: standard error of mean.

Creatinine and blood urea nitrogen (BUN) were significantly lower in the Hepa Wash group than in the control group at T12 (1.3 ± 0.2 vs. 3.2 ± 0.3, p=0.01 and 5 ± 0 vs. 10 ± 1, p=0.006, respectively). Ammonia (Figure 5) was effectively removed from the blood of the animals in the Hepa Wash group (562 ± 124 vs. 1382 ± 92 μg/dl, at T12, p=0.006). Nitrate/nitrite levels were similarly lower in the Hepa Wash group (5.54 ± 1.57 vs. 49.82 ± 13.27 μmol/l at T12, p=0.01). Significant differences between the Hepa Wash and the control groups were found for blood pH and glucose (Table 2).

**Table 2:** List of the main biochemical and hematological parameters of animals in the control and Hepa Wash groups

**Time [h]**	**T0**			**T4**			**T8**			**T12**	
**Group**	**Controls (n=5)**	**Hepa Wash (n=6)**	**MARS (n=2)**	**Control (n=5)**	**Hepa Wash (n=6)**	**MARS (n=2)**	**Control (n=5)**	**Hepa Wash (n=6)**	**MARS (n=2)**	**Control (n=2)**	**Hepa Wash (n=6)**
Blood pH	7.41±0.02	7.42±0.02	7.44, 7.41	7.35±0.03	7.41±0.02	7.35, 7.34	7.33±0.02	7.44±0.02*	7.36,7.33	7.27, 7.30	7.42±0.01*
Sodium [mmol/l]	136±1	136±1	133, 136	137±1	138±2	132, 134	134±1	143±2*	130, 130	138, 133	142±2*
Potassium [mmol/l]	4.3±0.1	4.4±0.2	4.9, 4.3	4.4±0.2	4.5±0.3	5.1, 4.23	4.8±0.2	4.0±0.2	4.9, 5.2	5.4, 5.7	4.4±0.3
Glucose [mg/dl]	98±9	112 ± 8	95, 107	111±14	109±10	143, 135	154±48	106±3	102, 145	120, 127	97±6*
ALP [U/l]	130±12	128 ± 8	181, 101	199±12	187±13	277, 136	279±12	253±11	382, 226	304, 326	323±14
GPT [U/l]	27 ± 2	36 ± 2	37, 39	74 ± 15	152 ± 42	184, 83	111 ± 15	213 ± 26*	201, 226	128, 108	222 ± 19*
GOT [U/l]	29 ± 1	33 ± 4	17,28	962 ± 263	2607 ± 884	3086, 1179	1977 ± 348	4076 ± 446*	3464, 5593	2024, 2254	4250 ± 282*
Total protein [g/dl]	5.4±0.3	5.3 ± 0.2	5.8, 5.6	5.2±0.3	5.2±0.1	5.2, 5.0	4.8±0.4	5.2±0.2	5.1, 4.9	4.3, 3.5	4.9±0.3
Total Bilirubin [mg/dl]	0.2±0.0	0.2±0.0	0.3, 0.2	0.6±0.1	0.5±0.1	0.7, 0.3	0.8±0.2	0.5±0.1	0.9, 0.5	1.0, 0.5	0.6±0.0
BUN [mg/dl]	9±1	9±1	11, 11	9±1	7±1	11, 9	9±1	5±0*	10, 10	8, 9	5±0.3*
Creatinine [mg/dl]	1.3 ± 0.1	1.3 ± 0.1	1.3, 1.1	1.4 ± 0.1	1.2 ± 0.1	1.5, 1.2	2.2 ± 0.1	1.0 ± 0.0*	2.1, 1.6	3.5, 3.5	1.3 ± 0.2*
Ammonia [μg/dl]	201 ± 31	178 ± 18	168, 235	514 ± 69	535 ± 61	734, 820	977 ± 193	454 ± 102	1072, 1077	1395,1268	562 ± 124*
Lactate [mmol/l]	2.0±0.1	1.6±0.1	1.2, 1.2	2.5±0.4	2.4±0.4	2.2, 1.6	1.9±0.1	1.9±0.2	1.5, 1.5	2.5, 3.5	2.5±0.4
INR	0.9±0.0	1.0±0.0	0.9, 0.9	1.2±0.1	1.3±0.1	1.1, 1.1	2.0±0.5	1.7±0.2	1.4, 1.9	1.7, 1.9	2.0±0.1
Fibrinogen [mg/dl]	425±77	456±18	513, 507	393±69	348±32	377, 372	303±71	262±21	290, 312	205,182	218±18
Hemoglobin [g/dl]	9.5±0.5	8.8±0.4	10, 10.4	10.1±0.5	8.1±0.5	9.1, 9.2	8.5±0.5	7.3±0.4	7.1, 8.3	7.8, 7.1	6.5±0.3
Platelets [G/l]	360±32	316±32	269, 291	279±51	196±19	180, 201	228±31	129±6	122, 130	104, 180	117±11
Antithrombin III [%]	65±3	70±2	83, 78	53±3	55±4	59, 55	42±2	42±2	47, 42	30, 31	33±1
Nitrate/Nitrite [μmol/l]	25.4±7.6	33.5±10.7	-	22.8±6.5	27.8±7.9	-	29.4±8.7	7.0±2.0	-	33.76, 76.15	5.5±1.6

*BUN*: blood urea nitrogen. *ALP*: alkaline phosphatase.*p<0.05 (difference between Hepa Wash and control).

### Safety of Hepa Wash procedure

No significant differences were seen between the two groups for platelets or other coagulation parameters like antithrombin-III (Table 2). Despite anticoagulation with heparin in both groups no purpural skin lesions or internal hemorrhage was observed during autopsy.

## Discussion

This preclinical study evaluated a new type of albumin dialysis, the Hepa Wash procedure. The extracorporeal procedure was used for the first time in the treatment of pigs with ALF. The ischemic model used in this study was described in a previous work [18] and has a relatively large therapeutic window with less pronounced elevation of intracranial pressure in comparison with other surgical models used to evaluate other liver support systems, where the animals were smaller in size and the portal vein diversion was established by an end-to-side portacaval shunt [21-24]. Avoiding splanchnic congestion and severe hypotension during surgery by performing side-to-side portacaval shunt instead prevents the development of a multisystem organ failure at an earlier stage and produces only a moderate elevation of ammonia. Though cerebral edema is not a prominent feature of this ALF model, it still corresponds to the clinical situation in the majority of cases, where the overall incidence of clinical cerebral edema in association with ALF appears to be decreasing (less than 25%), while the incidence of multiple organ failure as a mode of death is increasing [25,26].

Importantly, the ALF model used allows the evaluation of liver support systems, especially with the elevated levels of protein-bound and water-soluble toxins and the presence of multiple organ failure. We noticed many beneficial effects of the Hepa Wash in this model. Treatment enhanced cardiovascular stability and prevented the decline of cardiac index seen in the control group. This stabilization of cardiac index could be due to the removal of NO [27,28], which may lead to disturbances of the myocardial contractility [29,30].

The use of invasive measurement of the cardiovascular system (PiCCO System) helped us to eliminate difference in fluid balances as confounding factor. Early and continuous adjustment of GEDI and ELWI in both groups excludes volume depletion in the control group, where further elevation of GEDI by fluid infusions would worsen the already high ELWI values. Though they are part of the standard medical therapy [19], we did not use vasopressors in order to avoid the addition of a confounding factor, that may make the interpretation of the results more difficult.

The preserved urine production in the Hepa Wash group might additionally have contributed to the lower creatinine and BUN values, as these water soluble toxins were also effectively eliminated by Hepa Wash. A criticism to the design of the studies may emerge as the medical management of control animals did not comprise hemodialysis. Hyperkalemia was treated successfully with insulin-glucose therapy. No hypoglycemia was observed which could have been a confounding factor for the worse outcome in the control group. The metabolic acidosis was not severe enough to warrant hemodialysis. Oliguria in the ALF model was the only clinical condition which might have prompted the initiation of hemodialysis but then the mean arterial pressure was already very low to allow for treatment with extracorporeal procedures [31].

The improvement of the cerebral perfusion pressure in the treatment groups was mainly due to amelioration of the mean arterial pressure since the intracranial pressure in the ALF animal model did not show rapid and severe elevations. Therefore, cerebral perfusion pressure appears to be a better parameter to assess the treatment effects. These more pronounced effects on cerebral perfusion pressure are supported by elimination of ammonia by Hepa Wash. In the animal studies on MARS and Prometheus [21,22], the intracranial pressure was significantly reduced, though no significant elimination of ammonia was found. The lower levels of intracranial pressure in our ALF model compared with the other models and the resultant difficulty in showing significant differences may explain the different results.

The treatment with Hepa Wash may have improved survival by supporting the detoxification function of the liver and the kidney, thereby interrupting the vicious cycle of elevated toxin level and the resultant worsening of multiple organ failure. Removal of liver disease-related toxins like ammonia, creatinine, bilirubin and vasodilators may reduce their toxic effects and improve the multiple organ failure. We did not use scoring systems like SOFA or MELD as they should be first validated in this animal model (which is of a short duration). The use of surrogate markers and mortality may obviate the use of scoring systems. Bilirubin elimination was not measured in the dialysate or filtrate, whereas the removed acid–base from blood is difficult to accurately quantify due to the nature of the procedure which involves adding strong acid and base to the dialysate. However, the improvement of plasma levels of these surrogate markers may support our assumption of the good detoxification capacity of the procedure.

The MARS system was able to improve the hemodynamic instability before the sudden and severe elevation of intracranial pressure has ensued. Why this elevation occurred only in the MARS animals and not in the Hepa Wash group is not clear but it may be related to the lower capacity of the procedure to remove ammonia and the resultant cerebral edema. In any case, the results are in line with the principle of limited available space and the normal exponential pressure-volume relationship of the cranium as described by Marmour et al. [32].

The authors believe that Hepa Wash could offer many advantages over other artificial liver support systems. In contrast to MARS or Prometheus where clearance of toxins significantly declines after the first two hours due to saturation of adsorbents or anion exchangers [33,34], the Hepa Wash circuit has two conventional hemofilters which represent the site for eliminating the toxins. The albumin dialysate has an albumin concentration of 2% i.e. one tenth the concentration of albumin in the MARS procedure (20%). The efficient removal of toxins through passage in the Hepa Wash circuit allows the conduct of the dialysis procedure at higher flow rates than those used routinely in the MARS or the SPAD procedures. In the Hepa Wash procedure, the dialysate flow in the blood dialyzer can be increased to 60 l/h in comparison to 1 l/h in the SPAD procedure [5,6]. Plasma levels of different medications including those that are protein bound may be affected in this relatively non-selective elimination procedure. Threrefore, close monitoring and substitution according to needs is required. Improved and consistent detoxification of both protein-bound and water soluble toxins were supported with safety aspects. The Hepa Wash procedure did not cause adverse events and did not result in bleeding despite the direct measurement of the intracranial pressure and the presence of several fresh surgical wounds like the laparotomy wound. The Hepa Wash, however, substitutes only some of the liver and kidney detoxification functions without replacement of synthetic functions which requires administration to the patient (e.g. coagulation factors).

## Conclusions

The authors admit that these are only preclinical results with only a small sample size. We believe despite these limitations that the preliminary animal data of biochemical, organ function and survival improvement by the Hepa Wash are encouraging. Providing an adequate liver dialysis dose by increasing the capacity for the elimination of water-soluble and protein-bound toxins could enhance the efficacy of artificial liver systems and halt multisystem organ failure. Patients with ALF may show an improvement of mortality if they were treated early in the course of their illness with the new extracorporeal procedure. Clinical studies appear to be justified.

## Abbreviations

SPAD: Single pass albumin dialysis; MARS: Molecular adsorbent recirculating system; ALF: Acute liver failure; GEDI: Global enddiastolic volume index; ELWI: Extravascular lung water index; ACT: Activated clotting time; SVRI: Systemic vascular resistance index.

## Competing interests

Bernhard Kreymann is the Chief Executive Officer (CEO) of the company Hepa Wash GmbH and own stocks in the company. Ahmed Al-Chalabi own stocks/options in Hepa Wash GmbH (<1%). Catherine Schreiber, Patrick Schimmel and Ewald Nairz are employed by Hepa Wash GmbH and own stocks/options in the company (<1%). Other authors declare that they have no conflict of interests.

## Authors’ contributions

AA participated in the conception and design of the study, performed the surgical procedures, drafted the manuscript and performed the statistical analysis. EM performed the surgical procedures, helped to draft the manuscript. AT anesthetized, prepared and monitored animals and performed surgical procedures. PL arranged biochemical analysis of samples and revised manuscript. AP performed histopathological analysis. AN was involved in revising the manuscript especially the statistical analysis and the interpretation of results. TS participated and revised the statistical analysis and interpretation of data. CS, PS, EN and ZY prepared and operated the new liver support system. PR, WH and RMS revised and improved the manuscript. BK helped in drafting the manuscript and participated in the conception and design of the study as well as in the interpretation of data. All authors read and approved the final manuscript.

## Pre-publication history

The pre-publication history for this paper can be accessed here:

http://www.biomedcentral.com/1471-230X/13/83/prepub
